# Functional Assessment of 2,177 U.S. and International Drugs Identifies the Quinoline Nitroxoline as a Potent Amoebicidal Agent against the Pathogen *Balamuthia mandrillaris*

**DOI:** 10.1128/mBio.02051-18

**Published:** 2018-10-30

**Authors:** Matthew T. Laurie, Corin V. White, Hanna Retallack, Wesley Wu, Matthew S. Moser, Judy A. Sakanari, Kenny Ang, Christopher Wilson, Michelle R. Arkin, Joseph L. DeRisi

**Affiliations:** a Department of Biochemistry and Biophysics, University of California, San Francisco, San Francisco, California, USA; b California State University Monterey Bay, Seaside, California, USA; c Department of Pharmaceutical Chemistry, University of California, San Francisco, San Francisco, California, USA; d Small Molecule Discovery Center, University of California, San Francisco, San Francisco, California, USA; e Chan Zuckerberg Biohub, San Francisco, California, USA; Princeton University; CDC; McGill University

**Keywords:** amoeba, antiparasitic agents, balamuthia, encephalitis, nitroxoline

## Abstract

Balamuthia mandrillaris is a pathogenic free-living amoeba that causes a rare but almost always fatal infection of the central nervous system called granulomatous amoebic encephalitis (GAE). Two distinct forms of B. mandrillaris—a proliferative trophozoite form and a nonproliferative cyst form, which is highly resistant to harsh physical and chemical conditions—have been isolated from environmental samples worldwide and are both observed in infected tissue. Patients suffering from GAE are typically treated with aggressive and prolonged multidrug regimens that often include the antimicrobial agents miltefosine and pentamidine isethionate. However, survival rates remain low, and studies evaluating the susceptibility of B. mandrillaris to these compounds and other potential therapeutics are limited. To address the need for more-effective treatments, we screened 2,177 clinically approved compounds for *in vitro* activity against B. mandrillaris. The quinoline antibiotic nitroxoline (8-hydroxy-5-nitroquinoline), which has safely been used in humans to treat urinary tract infections, was identified as a lead compound. We show that nitroxoline inhibits both trophozoites and cysts at low micromolar concentrations, which are within a pharmacologically relevant range. We compared the *in vitro* efficacy of nitroxoline to that of drugs currently used in the standard of care for GAE and found that nitroxoline is the most potent and selective inhibitor of B. mandrillaris tested. Furthermore, we demonstrate that nitroxoline prevents B. mandrillaris-mediated destruction of host cells in cultured fibroblast and primary brain explant models also at pharmacologically relevant concentrations. Taken together, our findings indicate that nitroxoline is a promising candidate for repurposing as a novel treatment of B. mandrillaris infections.

## Abstract (precis)

Balamuthia mandrillaris is responsible for hundreds of reported cases of amoebic encephalitis, the majority of which have been fatal. Despite being an exceptionally deadly pathogen, B. mandrillaris is understudied, leaving many open questions regarding epidemiology, diagnosis, and treatment. Due to the lack of effective drugs to fight B. mandrillaris infections, mortality rates remain high even for patients receiving intensive care. This report addresses the need for new treatment options through a drug repurposing screen to identify novel B. mandrillaris inhibitors. The most promising candidate identified was the quinoline antibiotic nitroxoline, which has a long history of safe use in humans. We show that nitroxoline kills B. mandrillaris at pharmacologically relevant concentrations and exhibits greater potency and selectivity than drugs commonly used in the current standard of care. The findings that we present demonstrate the potential of nitroxoline to be an important new tool in the treatment of life-threatening B. mandrillaris infections.

## INTRODUCTION

The opportunistic protist pathogen Balamuthia mandrillaris causes rare but life-threatening infections of the central nervous system (CNS), termed balamuthia or granulomatous amoebic encephalitis (GAE) (1, 2). Onset of the disease is gradual and chronically develops over a few weeks to months in both immunocompromised and immunocompetent individuals worldwide (1, 3). Presenting clinical symptoms include but are not limited to fever, vomiting, neck stiffness, headache, nausea, personality changes, and seizures (1, 3). These symptoms are nonspecific and overlap symptoms caused by more common brain infections such as bacterial and viral meningitis as well as noninfectious neuroinflammatory syndromes. Cutaneous presentation is less common and can produce symptoms ranging from painless swelling to ulceration and formation of large lesions (4–6). Infections involving other organs ranging from the lungs to the eye have also been documented (7, 8). Thus, B. mandrillaris-induced encephalitis often goes unrecognized and diagnosis is frequently only made postmortem. Several hundred cases have been reported; however, the actual disease burden is likely underestimated (2, 9).

While systematic ecological studies of B. mandrillaris have not been performed, free-living B. mandrillaris amoebae have been isolated from water, soil, and dust across all continents (10–18). Cases of human and animal infections are also reported on all continents but are most common in South America and the southern United States (19–35) (reviewed in references 31, 32, and 33). B. mandrillaris is thought to be transmitted by inhalation of contaminated aerosols or exposure via broken skin (36). Fatal amoebic encephalitis has also occurred after solid-organ transplantation (37, 38). Pathogenesis is believed to involve hematogenous spread to the CNS through penetration of the blood-brain barrier, and amoebae are frequently observed around blood vessels (3, 36).

Free-living amoebae such as Sappinia pedata*, Acanthamoeba* spp., and Naegleria fowleri can also cause infection of the CNS with very poor prognosis (2, 39). *Acanthamoeba* and *Balamuthia* are the most similar and are classified in the same eukaryote supergroup (*Amoebozoa*: *Acanthamoebidae*) (40). The mode of infection employed by *Sappinia pedata* and *Acanthamoeba* spp. is thought to be similar to that used by *Balamuthia*, as encephalitis caused by these genera progresses over several weeks to months and is associated with water or soil contact. Acute encephalitis caused by N. fowleri is specifically associated with recreation in warm freshwater environments, with presumed neuroinvasion of the amoeba by passing up the nose through the cribiform plate to the brain (41). All of these pathogenic amoebae have a proliferative trophozoite form and a dormant, thick-walled cyst form, while N. fowleri also has a motile flagellate form. The cyst form of free-living amoebae is notoriously more resistant to antimicrobials (42–47) and to a variety of abiotic stressors such as UV light (48–50). Such attributes, along with the fact that drug sensitivities differ among genera, species, and strains of free-living amoebae, have complicated studies in drug discovery (51).

Infection of the CNS by B. mandrillaris is almost always fatal, and no specific and highly successful treatment regimen is known (45, 52). The CDC recommends the following drugs for treatment of B. mandrillaris CNS infection: pentamidine isethionate, miltefosine, fluconazole, flucytosine, sulfadiazine, azithromycin, and/or clarithromycin (53). *In vitro* studies performed with the CDC-recommended drugs have shown little to no inhibition of amoebic growth by fluconazole, sulfadiazine, and flucytosine, while azithromycin, pentamidine isethionate, miltefosine, and voriconazole (a fluconazole derivative) exhibit amoebicidal or amoebistatic activity (11, 42, 51). Current treatments for B. mandrillaris CNS infections employing experimental combinations of these drugs have produced inconsistent outcomes, including survival in some cases and fatality in others (7, 19, 23, 27, 29, 30, 34, 54–62). As the efficacy and specificity of current treatments remain uncertain, there is a clear need to identify additional drugs that can improve patient outcomes.

The goal of this study was to identify, from a set of clinically approved compounds, candidates that have the potential to be repurposed for treatment of B. mandrillaris infections. Here, we screened a library of 2,177 clinically approved compounds and found that the quinoline antibiotic nitroxoline (8-hydroxy-5-nitroquinoline) exhibits amoebicidal activity at low micromolar concentrations, well within the range of the estimated plasma concentrations achieved with recommended oral dosing (63–65). Through direct *in vitro* comparisons, we found that nitroxoline is a substantially more potent and selective inhibitor of B. mandrillaris than three commonly used GAE treatments currently recommended by the CDC. In addition to killing B. mandrillaris trophozoites, nitroxoline treatment also causes encystment and substantially delays recrudescence of active amoebae, which is significant considering the rapid decompensation of patients suffering from GAE. Nitroxoline, with its ease of delivery and favorable pharmacodynamic properties, has the potential to be used as an effective treatment for GAE in singularity or in combination with drugs in the current standard of care.

## RESULTS

### Identification of nitroxoline as an inhibitor of B. mandrillaris trophozoites *in vitro*.

**FIG 1:**
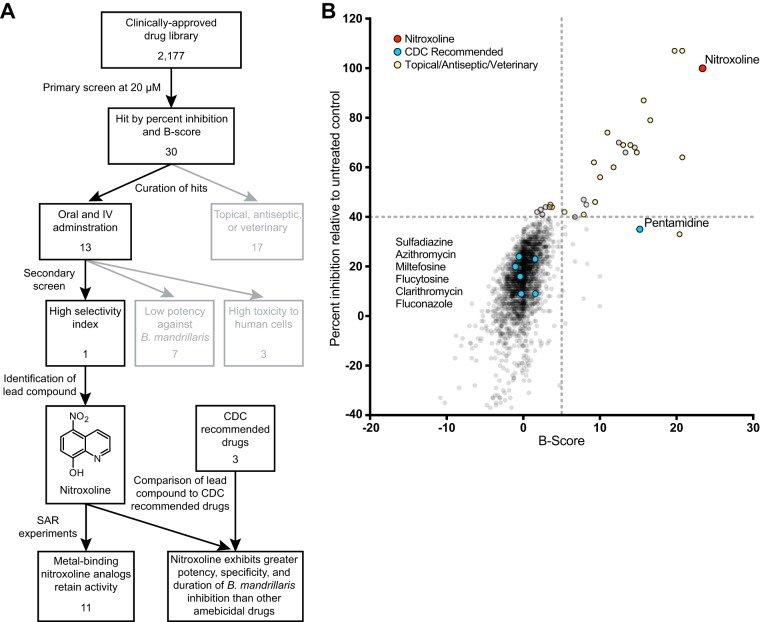
(A) Workflow for screening of clinically approved compounds for *in vitro* activity against B. mandrillaris. A primary screen of 2,177 clinically approved compounds yielded 30 hits meeting the percent inhibition and B-score criteria, among which only 13 candidates were available for oral or intravenous (IV) administration (see panel B). Secondary screening identified only one novel lead compound, nitroxoline, which displayed high selectivity for inhibition of B. mandrillaris viability (Table S1C). Structure-activity relationship (SAR) experiments showed that 11 of 12 nitroxoline analogs tested with potential metal binding domains remained active against B. mandrillaris, suggesting that metal binding plays a role in the mechanism of inhibition by nitroxoline (Fig. 2). Comparison of nitroxoline to three drugs recommended by the CDC for treatment of B. mandrillaris CNS infections (pentamidine isethionate, miltefosine, and azithromycin) indicates that nitroxoline is the most potent and specific inhibitor of B. mandrillaris among the compounds tested (Fig. 3 and 4; see also Table S1). (B) Plot of percent inhibition relative to untreated controls and B-score measured for each compound in a library of 2,177 clinically approved compounds. Raw data used to calculate these values are compiled in Table S1A. Drugs recommended by the CDC for treatment of GAE are highlighted in blue. Drugs that are classified as antiseptic or topical and/or have not been used in humans are shown in yellow. The quinoline antibiotic nitroxoline, which was the top hit identified in this screen, is highlighted in red.

Due to the extremely high mortality rate associated with Balamuthia mandrillaris infections and the limited efficacy of current treatments, there is a clear need to identify additional therapeutic strategies to improve patient outcomes in these rare but deadly infections. Here, we established replicating axenic cultures of B. mandrillaris (ATCC PRA-291) and screened 2,177 clinically approved compounds for reduction of trophozoite viability following 72 h of treatment at 20 µM. Compounds with high percent inhibition (∼40% or above) and B-score (∼5 or above) were nominated for secondary screening (Fig. 1A; see also Table S1A in the supplemental material). The remaining compounds were annotated by class and delivery method to eliminate drugs approved only for topical delivery or veterinary use, which have low therapeutic potential (Table S1B). The selected candidate drugs were tested in dose-response assays with B. mandrillaris trophozoites as well as HFF-1 and H4 human cell cultures to confirm their activity and evaluate toxicity to human cells (Table S1C). Half-maximal inhibitory concentration (IC_50_) values indicated that only two compounds, pentamidine isethionate and nitroxoline, demonstrated adequate potency against B. mandrillaris without high toxicity to human cells. Since the amoebistatic activity of pentamidine has previously been described (46), we focused on further characterizing the novel activity of nitroxoline as the lead compound identified by this screen.

10.1128/mBio.02051-18.4 TABLE S1 Tabulation of raw data from primary and secondary drug screens. (A) A primary screen of 2,177 clinically approved compounds measured the decrease in B. mandrillaris trophozoite viability resulting from 72 h of exposure to a 20 µM concentration of each compound. Viability was measured for test and control wells by the use of the CellTiter Glo luminescence assay. Raw luminescence intensity values for test wells are reported as well as the average luminescence intensity values for the positive and negative controls used for comparison. The B-score and percent inhibition shown for each compound were calculated using the raw luminescence intensity values. (B) List of compounds meeting loosely applied criteria of a B-score of approximately 5 or greater and inhibition of approximately 40% or greater. The class and delivery method are annotated for each compound. Compounds that are classified as veterinary or are available only for external/topical administration are highlighted in yellow. Nitroxoline, the top hit compound based on B-score and percent inhibition, is highlighted in red. (C) Secondary screening of hit compounds to determine potency against B. mandrillaris trophozoites and toxicity to HFF-1 and H4 human cell lines. All IC_50_ values were approximated from 8-point dose-response curves. IC_50_ values highlighted in red are outside a desirable range for a hit compound, as they indicate either low potency against B. mandrillaris (IC_50_ > 30 µM) or high toxicity to human cells (IC_50_ < 1 µM). Nitroxoline and pentamidine isethionate were the only compounds that inhibited B. mandrillaris trophozoite viability at concentrations similar to or lower than concentrations that were toxic to human cells. Download Table S1, XLSX file, 0.3 MB. Copyright © 2018 Laurie et al. 2018 Laurie et al. This content is distributed under the terms of the Creative Commons Attribution 4.0 International license.

### Nitroxoline structure-activity relationship (SAR) experiments.

**FIG 2:**
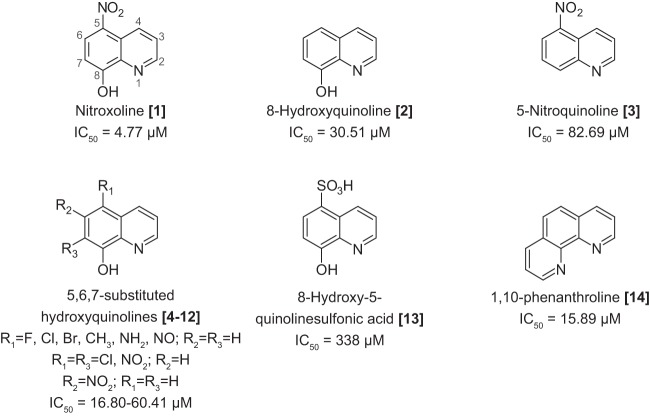
Structure-activity relationship experiments suggest that nitroxoline inhibits B. mandrillaris through a mechanism related to metal binding. Structures and IC_50_ values are shown for nitroxoline and select analogs; additional compounds are shown in Table S2. Nitroxoline is made up of a quinoline core with a nitro group at the 5 position and a hydroxyl group at the 8 position. Analogs lacking the 8-position hydroxyl group were generally inactive, with IC_50_ values greater than 80 µM (e.g., 5-nitroquinoline [compound 3]). Twelve nitroxoline analogs with predicted metal binding activity were tested, and of these, 1,10-phenanthroline (compound 14) and 10 of 11 compounds with an 8-position hydroxyl group (e.g., 8-hydroxyquinoline [compound 2]) were active, with IC_50_ values ranging from 17 to 60 µM. The only inactive analog with an 8-position hydroxyl group was 8-hydroxy-5-quinolinesulfonic acid (compound 13). Variance of the 5-position nitro group reduced potency compared to nitroxoline, but no trend related to aromatic electronic effects is apparent.

Nitroxoline is currently in clinical use as an antimicrobial drug in certain European and Asian countries. The hypothesized mechanism of action (MoA) is as a metal chelator that disrupts biofilm formation (66). Nitroxoline and other 8-hydroxyquinolines have also previously demonstrated *in vitro* anticancer activity, and free-radical metabolites are thought to be involved in cell death (67). To test which of these mechanisms is applicable in killing B. mandrillaris, we tested 19 commercially available analogs of nitroxoline for amoebicidal activity. While nitroxoline itself remained the most potent compound tested, there was a clear necessity for the 8-position hydroxyl group on the quinoline ring to retain high activity (Fig. 2; see also Table S2). As demonstrated by the activity of 8-hydroxyquinoline (compound 2), the 5-position nitro group is not necessary for activity, while in contrast, retaining the nitro group without the hydroxy group (compound 3) significantly decreases activity. Replacing the nitro group at the 5 position of the 8-hydroxyquinoline core with a variety of other functional groups, along with dual 5,7-position functionalization (compounds 4 to 13) results in several active but less potent analogs with no clear trend due to aromatic electronic affects. The sulfonic acid functionalized variant (compound 13) was the sole inactive compound among compounds 4 to 13, which we speculate may have been due to differences in uptake or permeability. Lastly, phenanthroline (compound 14), a structurally similar bidentate metal binding ligand, also demonstrated activity against B. mandrillaris. Taken together, these results indicate a likely metal binding mechanism for the original nitroxoline compound.

10.1128/mBio.02051-18.5 TABLE S2 Structures, IC_50_ values, and dose-response curve-fitting parameters for nitroxoline and all nitroxoline analogs tested for activity against B. mandrillaris. IC_50_, *R*^2^, and Hill slope values were calculated based on sigmoidal curves fitted to data from 8-point dose-response experiments performed in triplicate. Compounds 2, 4 through 12, and 14 were the most potent inhibitors of B. mandrillaris, most likely due to the presence of an 8-position hydroxyl group or bidentate ligand on the quinoline ring, each of which is predicted to be necessary for metal binding. Download Table S2, PDF file, 0.4 MB. Copyright © 2018 Laurie et al. 2018 Laurie et al. This content is distributed under the terms of the Creative Commons Attribution 4.0 International license.

### Direct comparison of nitroxoline to standard-of-care drugs for GAE treatment.

**FIG 3:**
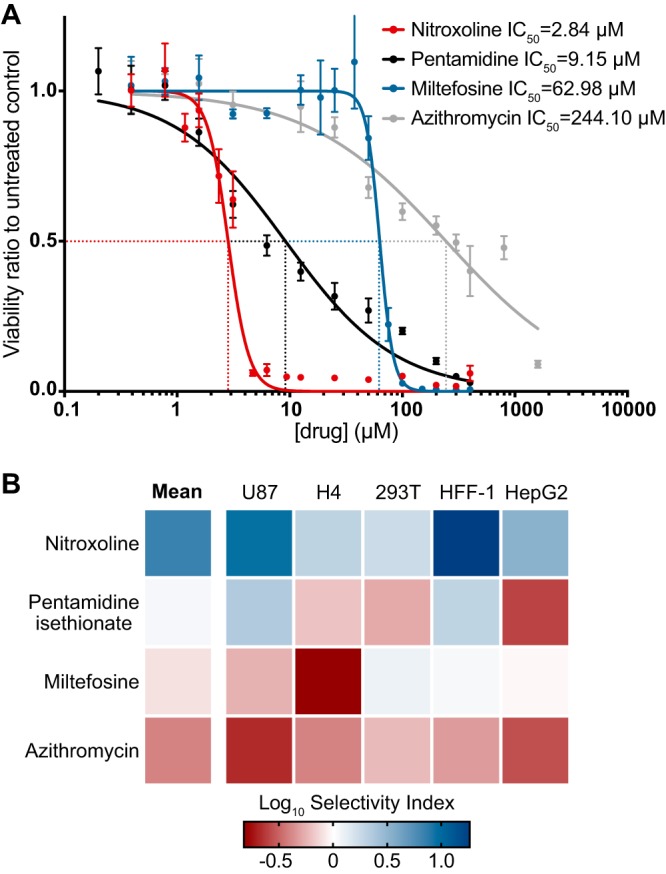
Potency and selectivity for inhibition of B. mandrillaris viability by nitroxoline, pentamidine isethionate, miltefosine, and azithromycin. (A) Dose-response curves show the effect of nitroxoline (red), pentamidine isethionate (black), miltefosine (blue), and azithromycin (gray) on the viability of B. mandrillaris trophozoite populations following 72 h of treatment. Data points represent means and standard errors of results from at least three independent biological replicates. Nitroxoline was the most potent inhibitor of B. mandrillaris viability, with an IC_50_ of 2.84 µM. (B) Heat map showing the Log_10_ selectivity index (human cell CC_50_/B. mandrillaris IC_50_) for nitroxoline, pentamidine, isethionate, miltefosine, and azithromycin calculated from the ratio of human cell CC_50_ to B. mandrillaris IC_50_. Nitroxoline exhibited the greatest mean Log_10_ selectivity index at 0.832 and was the only drug with a positive Log_10_ selectivity index (comparing B. mandrillaris inhibition to the results from all cell lines tested).

To evaluate nitroxoline as a potential drug to treat B. mandrillaris infections, we compared the *in vitro* performance of nitroxoline to that of pentamidine isethionate, miltefosine, and azithromycin, three drugs recommended by the CDC and commonly used in treatment of GAE (53). We performed side-by-side dose-response experiments to measure the efficacy of each drug against B. mandrillaris trophozoites and the toxicity to different human cell types using the following cell lines: HFF-1 (fibroblast), H4 (glial), U87 (glial), HEK-293T (kidney), and Hep-G2 (liver). Nitroxoline was the most potent inhibitor of B. mandrillaris trophozoites, with an IC_50_ of 2.84 µM compared to IC_50_ values of 9.14 µM, 63.23 µM, and 244.10 µM for pentamidine, miltefosine, and azithromycin, respectively (Fig. 3A). Nitroxoline was also the only drug with an IC_50_ against B. mandrillaris that was lower than the half-maximal cytotoxic concentration (CC_50_) for all cell lines tested. The average Log_10_ selectivity index (CC_50_ for human cell toxicity/IC_50_ for B. mandrillaris inhibition) of nitroxoline across all cell lines was 0.832, compared to 0.049, −0.102, and −0.409 for pentamidine, miltefosine, and azithromycin, respectively (Fig. 3B [summarized in Table S3]). Combinations of nitroxoline with miltefosine and with pentamidine isethionate showed generally additive inhibitory effects on B. mandrillaris viability, suggesting that nitroxoline can be combined with other amoebicidal drugs to produce greater inhibition (see Fig. S2 in the supplemental material).

10.1128/mBio.02051-18.6 TABLE S3 Summary of IC_50_, CC_50_, and selectivity index (SI) values calculated from dose-response experiments. IC_50_ and CC_50_ values were calculated from dose-response curves generated by measuring the viability of cultured cells 72 h after drug treatment. Cytotoxicity of compounds was measured in U87, H4, HEK-293T, HFF-1, and Hep-G2 cell lines. All experiments were performed in triplicate. SI values for each drug show the ratio of the CC_50_ value for a given cell line to the IC_50_ value for B. mandrillaris trophozoites. An SI value greater than 1 indicates that the drug inhibits B. mandrillaris trophozoites at lower concentrations than were seen with the human cell line used for comparison (i.e., has positive selectivity). Nitroxoline is the most potent inhibitor (lowest IC_50_) of both forms of B. mandrillaris and has the highest SI for all of the cell lines, with a mean SI of 6.79. Download Table S3, PDF file, 0.2 MB. Copyright © 2018 Laurie et al. 2018 Laurie et al. This content is distributed under the terms of the Creative Commons Attribution 4.0 International license.

### Encystment response of B. mandrillaris.

**FIG 4:**
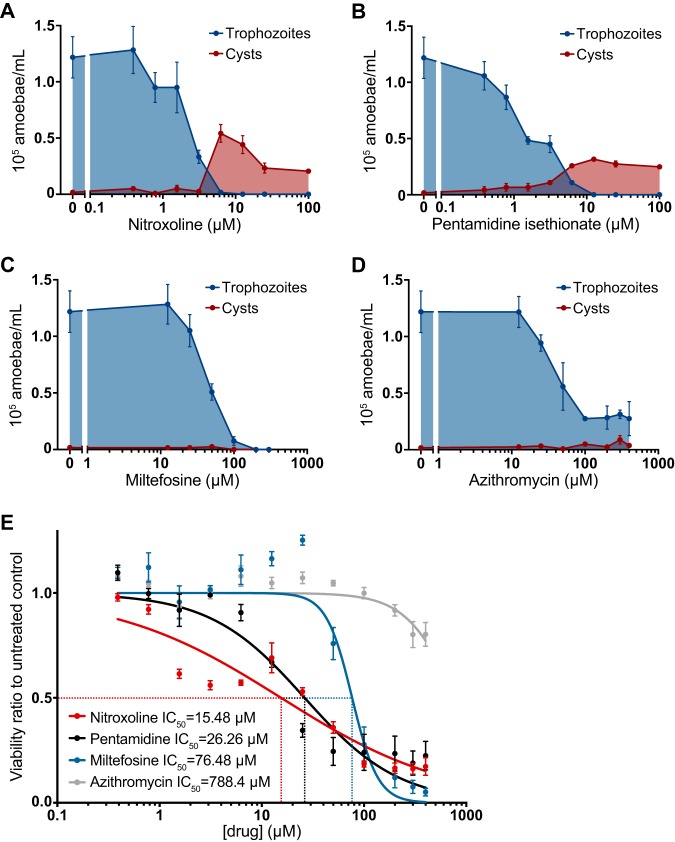
Relationship of drug treatment to B. mandrillaris encystment. (A to D) Changes in the number of trophozoites (blue) and cysts (red) in B. mandrillaris populations following 72 h of treatment with various concentrations of nitroxoline (A), pentamidine isethionate (B), miltefosine (C), and azithromycin (D). Low micromolar concentrations of nitroxoline and pentamidine isethionate caused an increase in the total number of cysts observed in B. mandrillaris populations and an increase in the ratio of cysts to trophozoites. No increase in encystment was observed in B. mandrillaris populations treated with miltefosine or azithromycin. (E) Dose-response curve showing the effect of nitroxoline (red), pentamidine isethionate (black), miltefosine (blue), and azithromycin (gray) on the viability of preformed B. mandrillaris cysts. Nitroxoline was the most potent inhibitor of cysts, with an IC_50_ of 15.48 µM. Cysts were substantially less sensitive than trophozoites (Fig. 2A) to all drugs except for miltefosine, which had similar IC_50_ values for inhibition of both B. mandrillaris forms.

In addition to a dose-dependent reduction in B. mandrillaris trophozoite viability, we also observed a general increase in the ratio of cysts to trophozoites correlated with increasing concentrations of some drugs. We investigated the propensity of nitroxoline, pentamidine isethionate, miltefosine, and azithromycin to induce encystment of B. mandrillaris by counting the number of cysts and trophozoites in culture samples following 72 h of treatment with different drug concentrations. We observed a dose-dependent reduction in the number of trophozoites in the population for all four drugs. Nitroxoline and pentamidine isethionate caused an increase in both the total number and the proportion of cysts in the population, while no substantial number of cysts was observed at any concentration of miltefosine or azithromycin (Fig. 4A to D). Because encystment appears to occur as a response to certain drug treatments, we also assessed the ability of each drug to inhibit the viability of preformed B. mandrillaris cysts. We induced encystment by sustained exposure to 12% galactose and conducted dose-response viability measurements for each drug. Nitroxoline was again the most potent inhibitor of cysts, with an IC_50_ of 15.48 µM compared to IC_50_ values of 26.26 µM, 76.48 µM, and 788.4 µM for pentamidine, miltefosine, and azithromycin, respectively (Fig. 4E). While nitroxoline, pentamidine, and azithromycin were considerably less potent inhibitors of cyst viability than of trophozoite viability, miltefosine inhibited the two forms of B. mandrillaris at similar concentrations (Table S3).

### Delayed recrudescence of B. mandrillaris treated with nitroxoline.

Nitroxoline and the tested standard-of-care drugs induce various combinations of distinct and intermediate B. mandrillaris phenotypes, including death and encystment. Though it is assumed that drug-induced phenotypes such as encystment affect the rate of amoeba population growth and host cell destruction, the magnitude and duration of these effects are unknown. To assess how rapidly amoeba populations recover and proliferate following drug treatment, we developed a recrudescence assay wherein we treated B. mandrillaris trophozoite cultures with various concentrations of nitroxoline, pentamidine, or miltefosine for 72 h, removed drug, and then added the remaining amoebae to a monolayer of HFF-1 cells in the absence of drug. The posttreatment recovery time was measured as the number of days required for each B. mandrillaris population to clear 100% of the host cells. Treatments that completely eliminated B. mandrillaris populations were determined by observing no live trophozoites or destruction of host cells at any point during the 28-day experiment. We found that 7 µM and 14 µM pentamidine delayed recovery of B. mandrillaris by 1 to 2 weeks but that increasing the dose from 14 µM to 56 µM delayed recovery by only an additional 3 days (Table S1). Consistent with the steep Hill slope observed in dose-response experiments, miltefosine caused very little delay in clearance time at 56 µM but completely eliminated B. mandrillaris populations at 112 µM. In contrast, nitroxoline delayed amoeba recovery by 2 to 3 weeks at low micromolar concentrations and completely eliminated B. mandrillaris populations at 28 µM.

### Protective effect of nitroxoline in a primary human brain tissue model.

**FIG 5:**
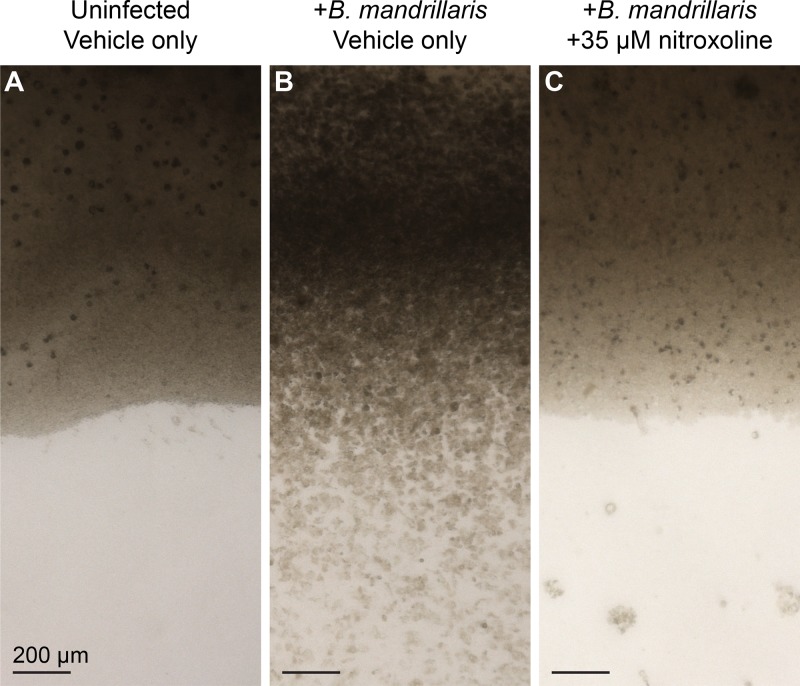
Nitroxoline prevents B. mandrillaris-mediated destruction of human brain tissue explants. Each panel shows an image representative of two tissues 96 h after exposure to the indicated conditions. Media were changed at 20 h postinfection to remove nitroxoline or vehicle. (A) Uninfected, untreated (vehicle only) tissues had distinct edges and maintained cell density throughout culture. (B) B. mandrillaris-infected, untreated (vehicle only) tissues show widespread damage, particularly at edges, where loss of cell density and disorder of tissue structure are apparent. Large numbers of B. mandrillaris trophozoites are observed intermixed with human cells and outside the tissue (lower half of image). (C) B. mandrillaris-infected tissues treated with 35 μM nitroxoline simultaneously with inoculation do not show signs of tissue damage or loss of cell density and maintain distinct edges similar to those of uninfected tissues. Clusters of B. mandrillaris cysts are observed outside the boundaries of the tissue.

Findings from the recrudescence assays suggest that nitroxoline treatment may significantly impede destruction of host cells in the context of B. mandrillaris infection. To further explore this possibility, we performed experiments modeling B. mandrillaris infection and nitroxoline treatment in primary human brain tissue. Human cortical tissue slices were exposed to B. mandrillaris trophozoites and simultaneously treated with nitroxoline or vehicle (dimethyl sulfoxide [DMSO]) for 20 h before media were changed to remove drug or vehicle. Tissues were cultured for 4 days and then evaluated for damage by microscopic examination (Fig. 5; see also Fig. S3). Untreated tissues showed widespread damage following B. mandrillaris exposure, including loss of distinct tissue edges and reduction of cell density (Fig. 5B). Large numbers of highly motile B. mandrillaris trophozoites were present at the edges of tissues and were intermixed with human cells (see Movies S1 to S3 in the supplemental material). In contrast, nitroxoline-treated tissues did not show signs of B. mandrillaris-mediated destruction and appeared to be similar to uninfected tissues (Fig. 5A and C). Cysts with little to no motility were observed outside the boundaries of tissues (Movie S4). While these findings are qualitative, the large-scale differences in tissue morphology observed in this experiment are consistent with the possibility that nitroxoline has a protective effect on host tissue in the context of B. mandrillaris infection.

10.1128/mBio.02051-18.7 MOVIE S1 Representative movie showing the edge of an untreated (vehicle only), uninfected primary brain tissue section 4 days after the start of culture. No significant movement of cells is observed, and a clearly defined edge marks the border of the tissue. Download Movie S1, AVI file, 4.3 MB. Copyright © 2018 Laurie et al. 2018 Laurie et al. This content is distributed under the terms of the Creative Commons Attribution 4.0 International license.

10.1128/mBio.02051-18.8 MOVIE S2 Representative movie showing the edge of an untreated (vehicle only), B. mandrillaris-infected primary brain tissue section 4 days after inoculation of amoebae (2 min duration). The movie is representative of two biological replicates. Highly motile B. mandrillaris trophozoites are observed throughout the field of view, and the tissue edges are not discernible due to loss of host cell density. Download Movie S2, AVI file, 4.7 MB. Copyright © 2018 Laurie et al. 2018 Laurie et al. This content is distributed under the terms of the Creative Commons Attribution 4.0 International license.

10.1128/mBio.02051-18.9 MOVIE S3 Representative movie showing the edge of an untreated (vehicle only), B. mandrillaris-infected primary brain tissue section 4 days after inoculation of amoebae (10 min duration). The movie is representative of two biological replicates. Highly motile B. mandrillaris trophozoites are observed throughout the field of view, and the tissue edges are not discernible due to loss of host cell density. Download Movie S3, AVI file, 20.1 MB. Copyright © 2018 Laurie et al. 2018 Laurie et al. This content is distributed under the terms of the Creative Commons Attribution 4.0 International license.

10.1128/mBio.02051-18.10 MOVIE S4 Representative movie showing edges of nitroxoline-treated, B. mandrillaris-infected primary brain tissue section 4 days after inoculation of amoebae. The tissue was treated with 35 µM nitroxoline simultaneously with inoculation of amoebae. Nonmotile B. mandrillaris cysts are observed individually primarily at the edge of the tissue and outside the tissue. The tissue edges are intact and do not show signs of host cell destruction by B. mandrillaris. Download Movie S4, AVI file, 3.3 MB. Copyright © 2018 Laurie et al. 2018 Laurie et al. This content is distributed under the terms of the Creative Commons Attribution 4.0 International license.

## DISCUSSION

GAE caused by Balamuthia mandrillaris is almost always fatal (19–34). Because there is no established treatment for GAE, patients commonly receive experimental combinations of antimicrobial agents in aggressive and prolonged treatment regimens with mixed outcomes (7, 19, 23, 27, 29, 30, 34, 54–62). In the present study, we aimed to address the critical need for new treatments of GAE by identifying novel amoebicidal compounds. Because our goal was to identify candidate drugs with established safety and pharmacodynamic profiles, we chose to screen a library of 2,177 clinically approved compounds. Based on the criteria of B-score and percent inhibition of B. mandrillaris viability, we selected 12 compounds from the primary screen for follow-up screening (Fig. 1A; see also Table S1B in the supplemental material). Secondary screening eliminated all candidate compounds on the basis of low potency for inhibition of B. mandrillaris or high toxicity to human cells, with the exception of the quinoline antibiotic nitroxoline. Nitroxoline demonstrated promising potency and selectivity for B. mandrillaris inhibition, which led us to focus our efforts on further investigating its novel amoebicidal activity.

We performed side-by-side experiments to directly compare the *in vitro* efficacy of nitroxoline with respect to B. mandrillaris inhibition to the efficacy of pentamidine isethionate, miltefosine, and azithromycin, three drugs recommended by the CDC for treatment of GAE and commonly used in case reports (29, 30, 53–55, 57). We found that nitroxoline was the most potent inhibitor of B. mandrillaris trophozoite viability, with an IC_50_ of 2.84 µM and IC_99_ of 7.54 µM (Fig. 3A). To estimate the selectivity of each drug, we also measured the toxicity of each compound to five different human cell lines. For pentamidine, miltefosine, and azithromycin, drug concentrations that reduced B. mandrillaris viability also caused significant toxicity to human cells, giving very small selectivity indices (Fig. 3B). In contrast, nitroxoline had an IC_50_ for B. mandrillaris inhibition that was lower than the CC_50_ for all cell lines tested and had the highest mean selectivity index value (Fig. 3B; see also Table S3).

B. mandrillaris and other free-living amoebae have a dormant, thick-walled cyst form which is highly resistant to several types of environmental stress, including exposure to some compounds that are toxic to the trophozoite form (44, 46, 48, 50). A previous study postulated that the transition from the trophozoite form to the cyst form can be triggered under a variety of conditions, including chemical stress (68), raising the possibility that treatment with amoebicidal or amoebistatic drugs may induce encystment. To investigate this possibility, we quantified the frequency of cysts observed in B. mandrillaris populations treated with different drug concentrations. We observed that low micromolar concentrations of nitroxoline and pentamidine isethionate caused increases in the total number and proportion of cysts in B. mandrillaris populations (Fig. 4A and B). In contrast, miltefosine and azithromycin were not observed to induce encystment at any concentration (Fig. 4C and D). These data support the possibility that B. mandrillaris encystment occurs as a response to some but not all compounds that are toxic to trophozoites. We suggest that compounds which promote encystment in addition to killing trophozoites may provide additional benefit in the context of infection by slowing or halting the rapid tissue destruction by trophozoites.

Because it is common for both cysts and trophozoites to be found in B. mandrillaris-infected tissue, it is important to understand the efficacy of amoebicidal drugs against both stages (69). To address this issue, we performed side-by-side dose-response experiments with homogenous populations of cysts or trophozoites treated with nitroxoline, pentamidine isethionate, miltefosine, and azithromycin. We found that cysts were less sensitive than the trophozoites to all four drugs (Table S3). The difference in sensitivity was marginal for miltefosine and greatest for nitroxoline, which had an IC_50_ for cyst inhibition 6-fold higher than the IC_50_ for trophozoite inhibition. Nevertheless, nitroxoline remained the most potent inhibitor of preformed B. mandrillaris cysts, with an IC_50_ of 15.48 µM.

Viability measurements of drug-treated B. mandrillaris cultures reflect a complex population phenotype that includes various degrees of both death and encystment. These measurements are not sufficient to predict the rate at which populations recrudesce following treatment, which is an important factor in evaluating the overall efficacy of different treatments. We chose to address this aspect of treatment efficacy by performing recovery assays in which B. mandrillaris populations were exposed to various drug treatments, removed from drug, and then cocultured with monolayers of human cells until the host cells were fully consumed. As predicted, increasing drug concentrations caused greater delays to B. mandrillaris recrudescence and host cell destruction (Table 1). Surprisingly, even low micromolar concentrations of nitroxoline near the IC_50_ for trophozoite inhibition delayed B. mandrillaris-mediated host cell destruction by 2 to 3 weeks. This assay also served as a sensitive method to detect very low numbers of surviving amoebae due to the large population expansion occurring over the 28-day experiment duration. Importantly, this sensitive assay allowed us to infer that B. mandrillaris populations had been completely eliminated by drug treatment when we observed no signs of amoeba population recovery after 28 days of coculture with host cells.

**TABLE 1:** Recrudescence time of active *B. mandrillaris* following drug treatment

Treatment	Mean no. of days for host cell clearance ± SE
Vehicle only	
0.28% (vol/vol) DMSO	1.33 ± 0.33
1.12% (vol/vol) water	1.67 ± 0.67
	
Nitroxoline (in DMSO)	
3.5 µM	15.33 ± 0.67
7 µM	19 ± 0.58
14 µM	22 ± 2.08
28 µM	NRT^*a*^
	
Pentamidine isethionate (in water)	
3.5 µM	1.33 ± 0.33
7 µM	7 ± 1.15
14 µM	13 ± 2.51
28 µM	15.33 ± 2.56
56 µM	16 ± 2
	
Miltefosine	
28 µM	1.5 ± 0.33
56 µM	3.5 ± 2.64
84 µM	>28^*b*^
112 µM	NRT^*a*^

a NRT, no recovery of trophozoites or host cell destruction observed through 28 days.

b Incomplete recovery of trophozoites and destruction of host cells on day 28.

Using this method, we determined that concentrations of 28 µM nitroxoline and 112 µM miltefosine completely eliminated B. mandrillaris populations. These data are consistent with viability inhibition experiments in indicating that nitroxoline is the most potent inhibitor of B. mandrillaris tested. The promising results of this experiment suggest that nitroxoline may be able to fully eliminate B. mandrillaris infection if sufficiently high concentrations are reached and is likely to cause substantial delays with respect to host tissue damage even at lower concentrations. This is supported by our findings indicating that nitroxoline prevented B. mandrillaris activity and tissue destruction in a primary human brain tissue model (Fig. 5; see also Movie S1 to S4 in the supplemental material). Together, these findings suggest that nitroxoline substantially impedes host tissue destruction by B. mandrillaris *in vitro*. Given the rapid progression of pathogenesis that is characteristic of GAE, any impediment to tissue destruction could significantly improve patient prognosis.

The known safety and pharmacokinetic properties of nitroxoline suggest further promise for its therapeutic value as an inhibitor of B. mandrillaris. Nitroxoline has been safely used for over 50 years in the treatment of urinary tract infections, with minimal adverse effects reported (70). Nitroxoline is available in oral and intravenous administration forms and is typically dosed at 600 to 800 mg/day for adults, resulting in maximal plasma concentrations (*C*_max_) of up to approximately 30 µM (5.6 mg/liter) (63–65), which is 10-fold higher than the IC_50_ for B. mandrillaris trophozoites *in vitro* (Fig. 3A; see also Table S3). Although the extent to which nitroxoline crosses the blood brain barrier is unknown, a recent study showed that systemically delivered nitroxoline exhibits efficacy against gliomas in mice, implying that efficacious concentrations reached the brain in that model (71). In addition, we previously noted that B. mandrillaris frequently causes necrotizing vasculitis in the CNS with extensive blood-brain barrier (BBB) breakdown, which may affect the bioavailability of systemically administered drugs in the CNS of patients actively suffering from GAE (3, 36, 57). Furthermore, given the extreme severity of GAE, intrathecal drug delivery can be performed to maximize drug concentrations reaching the brain (2, 72). While many variables may affect the *in vivo* efficacy of nitroxoline as well as the concentrations that can be achieved in relevant compartments, the literature suggests that the *in vitro* efficacious concentrations that we demonstrated are well within a pharmacologically relevant range.

The *in vitro* results presented in this study strongly indicate that nitroxoline warrants further investigation as a potential treatment for B. mandrillaris infections. As there were many variables that were untested in our study that may affect the efficacy of nitroxoline *in vivo*, future work will ideally focus on providing *in vivo* validation of the amoebicidal activity of nitroxoline. In particular, *in vivo* studies will determine if efficacious concentrations of nitroxoline reach the relevant tissues as well as provide comparative evidence for amoebicidal activity with respect to currently recommended compounds. We note that nitroxoline is an approved compound in several countries, with an established safety profile (70), and it is possible that it could be considered as an emergency experimental treatment in dire cases of GAE. In particular, since the current standard of care generally consists of experimental combinations of several antimicrobial agents, adding nitroxoline to these regimens may be a reasonable step in the effort to improve patient prognosis. Although our SAR experiments did not identify any analog that matched the potency of nitroxoline, medicinal chemistry optimization may still be beneficial for a better understanding of possible mechanisms of action and efforts to improve drug specificity. The similarity of B. mandrillaris to other free-living amoebae such as *Acanthamoeba* spp. and Naegleria fowleri raises the intriguing possibility that nitroxoline or related compounds may also have activity against these pathogens.

## MATERIALS AND METHODS

### Human cell lines.

Hep-G2 (ATCC HB-8065), U87 (gift of Jonathan Weissman, University of California, San Francisco [UCSF]), H4 (ATCC HTB-148), and HEK-293T (ATCC CRL-3216) cells were cultured in Dulbecco’s modified Eagle’s medium (Gibco) containing 10% (vol/vol) fetal bovine serum (FBS) (Gibco), 2 mM l-glutamine, 100 U/ml penicillin/streptomycin (Gibco), and 10 mM HEPES buffer. HFF-1 cells (ATCC SCRC-1041) were cultured in DMEM containing 15% (vol/vol) FBS, 2 mM l-glutamine, 100 U/ml penicillin/streptomycin, and 10 mM HEPES buffer (Gibco). All mammalian-cell incubation steps were carried out at 37°C with 5% CO_2_. All cell lines were tested for mycoplasma contamination using a Lonza Mycoalert mycoplasma detection kit (Lonza).

### Balamuthia mandrillaris propagation, handling, and encystment.

Balamuthia mandrillaris (ATCC PRA-291) was maintained axenically in 150-cm^2^ flasks (Corning) containing modified Cerva’s medium (axenic medium) with the following formulation: 20 g of Bacto Casitone (Difco), 68 ml of 10× Hanks’ balanced salt solution (Gibco), 10% fetal bovine serum, and 1× penicillin-streptomycin (200 IU/ml to 200 μg/ml) (73). Axenic growth of B. mandrillaris resulted in 2 × 10^5^ to 3 × 10^5^ amoebae/ml in log phase. Routine subculturing was performed every 3 to 4 days by transferring 5 ml of amoebae into 15 ml of fresh axenic media.

Following the findings of a previous study, encystment of B. mandrillaris was induced by galactose exposure (74). B. mandrillaris trophozoites were grown to log phase in axenic media, and galactose was added to reach a final concentration of 12% (vol/vol). Amoebae were cultured in the induction medium until trophozoites were no longer observed (approximately 10 days). Galactose-induced cysts transition back to trophozoites after approximately 3 days of incubation in galactose-free axenic medium. Therefore, all assays with cysts were completed in the induction medium. To quantify amoebae for use in experiments, actively growing trophozoites or recently induced cysts were centrifuged at 3,000 rpm for 5 min, resuspended in axenic medium, and counted with a disposable hemocytometer (SKC, Inc.). All incubation steps for B. mandrillaris growth were carried out at 37°C with 5% CO_2_.

### Primary drug screening of Balamuthia mandrillaris trophozoites *in vitro*.

Screening of a clinically approved library of compounds, compiled by the Small Molecule Discovery Center (SMDC) at the UCSF, was completed at 20 µM in 0.2% DMSO. All 2,177 drugs in the library were stored as 10 mM stocks dissolved in 100% DMSO (Sigma-Aldrich) at −20°C. B. mandrillaris amoebae were resuspended in axenic medium and distributed into opaque 384-well plates (Corning) at a density of 3,000 amoebae per well using a BioMek NX liquid handler. Negative-control wells were treated with vehicle only (0.2% DMSO in axenic medium), and positive-control wells simulating total destruction of amoebae were seeded with amoeba lysate generated by 3 consecutive freeze-thaw cycles. Following 72 h of incubation, 30 µl of CellTiter Glo reagent (CTG; Promega) was added to each well. The luminescence was measured with a Promega GloMax-Multi+ plate reader at 2, 4, and 8 h after CTG addition. The percent inhibition of B. mandrillaris was calculated based on the CTG luminescence measurements of treated wells relative to both positive and negative controls using the following equation: percent inhibition = 100 − 100*[(test well intensity − positive-control intensity)/(negative-control intensity − positive-control intensity)]. The B-score, representing a plate-based statistical approach for correcting row, column, and edge effects, was also calculated for each compound in the library (75). Raw luminescence measurements and computed inhibition values are displayed in Table S1A in the supplemental material. Hits represented compounds with a B-score of approximately 5 or above and percent inhibition of approximately 40% or above (Table S1B). Hit compounds that are approved only for topical administration or veterinary use were not tested in secondary screening (highlighted in yellow in Table S1B and Fig. 1B). Compounds identified in the primary screen that did not exhibit activity upon repurchase of fresh compound were removed from consideration in the screening funnel.

### Secondary drug screening against B. mandrillaris trophozoites and HFF-1 and H4 human cells *in vitro*.

Secondary screening of hit compounds consisted of dose-response experiments to measure the toxicity of each compound to B. mandrillaris trophozoites, HFF-1 human fibroblast cells, and H4 human neuroglioma cells. Four 384-well plates were prepared prior to addition of drug as follows: one opaque plate (Corning) seeded with 3,000 HFF-1 cells per well in 60 µl complete media, one opaque plate seeded with 2,000 H4 cells per well in 60 µl complete media, one opaque plate containing 30 µl axenic media, and one clear plate (Corning) containing 30 µl axenic media. Opaque plates were used to measure the viability of trophozoites in the CTG assay, and clear plates were used for microscopic examination of cyst formation. HFF-1 and H4 plates were seeded 24 h prior to drug addition. The selected primary screen hits were added from 10 mM DMSO stocks into wells of all four test plates to reach final concentrations ranging from 0.06 µM to 30 µM (10 concentrations, 2-fold serial dilution). Negative-control wells received concentrations of DMSO corresponding to the amount of DMSO in each tested drug well (reaching a maximum of 0.3% DMSO). B. mandrillaris trophozoites were resuspended in axenic media and added to the plates containing drug dilutions in axenic media at a density of 3,000 amoebae per well in a final volume of 60 µl media. All plates were incubated for 72 h. Throughout the incubation period, the clear-bottom B. mandrillaris plate was monitored for large-scale changes to population encystment in response to drug treatment. After the 72-h incubation, 30 µl of CTG reagent was added to all wells of the opaque assay plates and luminescence was measured with a Promega GloMax-Multi+ plate reader at 2, 4, and 8 h after CTG addition. IC_50_ values for inhibition of B. mandrillaris viability were determined using the GraphPad Prism 4-parametric sigmoidal curve-fitting model, with the bottom and top constraints set to 0 and 1, respectively (Table S1C).

### Nitroxoline structure-activity relationship experiments.

Nineteen commercially available analogs of nitroxoline were selected for structure-activity relationship (SAR) experiments based on variances in functional groups that may play a role in the observed mechanism of action (compound sourcing and data are presented in Table S2). B. mandrillaris was grown to log phase axenically and plated at 4,000 amoebae per well in opaque 96-well plates. Each nitroxoline analog was dissolved in 100% DMSO at 10 mM. Analog stocks were serially diluted in water to generate 8-point dilution series, which were then used to treat assay wells containing B. mandrillaris trophozoites at final concentrations ranging from 0.14 µM to 300 µM (8 concentrations, 3-fold serial dilution) in a final volume of 100 µl. After incubation for 72 h, 50 µl of CTG was added to all assay wells. Luminescence was measured using a Promega GloMax Multi+ luminometer 2, 4, and 8 h after CTG addition. IC_50_ values were determined using the GraphPad Prism 4-parametric sigmoidal curve-fitting model with bottom and top constrains of 0 and 1, respectively.

### Dose-response experiments with HFF-1, H4, U87, Hep-G2, and HEK 293T cells and Balamuthia mandrillaris trophozoites and cysts.

B. mandrillaris trophozoites were seeded at 4,000 amoebae per well into opaque and clear-bottom 96-well plates (Corning). Homogenous populations of B. mandrillaris cysts generated by galactose induction were seeded at 4,000 amoebae per well into opaque 96-well plates. HFF-1 (fibroblast), H4 (glial), U87 (glial), HEK-293T (kidney), and Hep-G2 (liver) cells were seeded at 3,000 cells per well in opaque 96-well plates 24 h prior to addition of drug. Stocks of nitroxoline (Selleck Chemicals) were dissolved in 100% DMSO at 10 mM. Stocks of azithromycin (Selleck Chemicals), pentamidine isethionate (Selleck Chemicals), and miltefosine (Selleck Chemicals) were dissolved in water at 10 mM. Drug stocks were serially diluted in water to generate 12-point dilution series, which were then used to treat assay wells containing B. mandrillaris or human cells at final concentrations ranging from 0.39 µM to 400 µM in 100-µl total well volumes. Control wells were treated with vehicle (DMSO or water) at concentrations corresponding to the final vehicle concentrations in each drug dilution series. After incubation for 72 h, 50 µl of CTG was added to all assay wells. Luminescence was measured with a Promega GloMax-Multi+ plate reader 2, 4, and 8 h after CTG addition. All dose-response experiments were performed with at least three independent biological replicates. IC_50_ values were determined as previously described. To quantify encystment under treated and untreated conditions, amoebae in individual assay wells were resuspended thoroughly and the number of cysts and number of trophozoites in 10-µl samples were counted by the use of a hemocytometer (SKC, Inc.). Cysts and trophozoites are morphologically distinct, making them simple to distinguish with bright-field microscopy (example images are shown in Fig. S1 in the supplemental material). Encystment assays were performed with three independent biological replicates.

10.1128/mBio.02051-18.1 FIG S1 Example bright-field images of B. mandrillaris trophozoites and cysts. (A) Example images show B. mandrillaris trophozoites in log-phase growth. Trophozoites are pleomorphic and can be elongated or generally rounded, often with highly branched pseudopodia. (B) Example images show B. mandrillaris cysts induced by galactose exposure. Cysts are spherical and generally smaller in diameter than trophozoites and can have visibly distinct layers. Some cysts show signs of vacuolization that may be indicative of cell death. Download FIG S1, PDF file, 3.3 MB. Copyright © 2018 Laurie et al. 2018 Laurie et al. This content is distributed under the terms of the Creative Commons Attribution 4.0 International license.

10.1128/mBio.02051-18.2 FIG S2 Efficacy of nitroxoline in combination with miltefosine and pentamidine isethionate for inhibition of B. mandrillaris. (A and B) Heat maps of treatment effect (percent reduction of B. mandrillaris trophozoite viability relative to untreated control) across dose matrices for nitroxoline in combination with miltefosine (A) and pentamidine isethionate (B). The viability of B. mandrillaris trophozoite populations was measured following 72 h of drug treatment in three independent replicates. (C and D) Heat maps of excess effect over highest single agent (EOHSA) showing the additional effect of dose combinations beyond the effect of the most active single agent in the combination (EOHSA = *E*_combo_ − *E*_HSA_) across dose matrices for nitroxoline in combination with miltefosine (C) and pentamidine isethionate (D). For both drug pairs, mild antagonism (negative values colored in red scale) was observed for a small number of combinations, whereas most combinations produced a modest additive effect (positive values colored in blue scale). No strong antagonism or synergy was observed between nitroxoline and miltefosine or pentamidine isethionate. Download FIG S2, PDF file, 0.3 MB. Copyright © 2018 Laurie et al. 2018 Laurie et al. This content is distributed under the terms of the Creative Commons Attribution 4.0 International license.

10.1128/mBio.02051-18.3 FIG S3 (A and B) Bright-field images representative of two biological replicates for human brain tissue explants before and after exposure to the indicated conditions. Nitroxoline or vehicle was added simultaneously with B. mandrillaris trophozoites and removed after 20 h. Four days after exposure to B. mandrillaris, untreated tissues showed widespread damage and loss of cell density whereas nitroxoline-treated tissues remained intact and appeared similar to uninfected tissues. Large numbers of B. mandrillaris trophozoites can be seen at the edges of untreated tissues, while only clusters of cysts are observed in nitroxoline-treated tissues. (C) Images representative of two biological replicates of human brain tissue explants fixed and stained with DAPI 4 days after exposure to the indicated conditions (two images per condition). The number of host cell nuclei was dramatically reduced in untreated, B. mandrillaris-infected tissues compared to uninfected tissues, whereas B. mandrillaris-infected tissues treated with nitroxoline show no apparent loss of nuclei. Download FIG S3, PDF file, 0.5 MB. Copyright © 2018 Laurie et al. 2018 Laurie et al. This content is distributed under the terms of the Creative Commons Attribution 4.0 International license.

Experiments measuring the effect of drug combinations were carried out similarly to the dose-response experiments described above. B. mandrillaris trophozoites were seeded into opaque 96-well plates (Corning) at a density of 4,000 amoebae per well and then treated with concentrations of nitroxoline that differed across the rows of each plate and with concentrations of either miltefosine or pentamidine isethionate that differed down the columns to generate 7-by-7 dose combination matrices. Final drug concentrations ranged from 0.5 to 10 µM, from 10 to 100 µM, and from 1 to 100 µM for nitroxoline, miltefosine, and pentamidine isethionate, respectively. After incubation with drug or vehicle was performed for 72 h, CTG was added to all assay wells and luminescence was measured with a Promega Glomax-Multi+ plate reader. The percent effect of each drug combination was calculated as the reduction in luminescence value relative to vehicle-only controls. The additive effect of drug combinations was assessed using the excess over highest single agent (EOHSA) model (76, 77). EOHSA values were calculated using the equation EOHSA = *E*_combo_ − *E*_HSA_, where *E*_combo_ is the percent effect of a given dose combination and *E*_HSA_ is the percent effect of the most active (highest) single agent in the drug pair. Drug combination experiments were performed with three independent replicates.

### Balamuthia mandrillaris recrudescence assays.

Populations of B. mandrillaris were diluted to 2.5 × 10^5^ amoebae in 10 ml media and were treated with 3.5, 7, 14, 28, 56, 84, and 112 μM nitroxoline, pentamidine isethionate, or miltefosine and incubated for 72 h. Following incubation, all remaining amoebae in each population (various mixtures of cysts and trophozoites) were pelleted at 3,000 rpm for 5 min and resuspended in drug-free HFF-1 medium. Each resuspended B. mandrillaris population was placed on a monolayer of HFF-1 cells that were seeded at 10^6^ cells per flask in 10 ml at 24 h prior to inoculation. At that cell density, untreated amoebae typically consume 100% of HFF-1 monolayers within 24 h. Coculture flasks containing amoebae and HFF-1 cells were incubated until 100% of the HFF-1 monolayer was consumed as observed by daily microscopic inspection or until the predetermined endpoint of the experiment at 28 days post-B. mandrillaris inoculation was reached. The day on which complete clearance of the HFF-1 monolayer occurred was recorded for all conditions. All recrudescence assays were performed with three independent biological replicates. These methods were adapted from a minimum trophozoite amoebicidal concentration (MTAC) assay that was conducted with monolayers of MA104 monkey kidney cells (42).

### Primary brain tissue model.

Deidentified tissue samples were collected with previous patient consent in strict observance of the legal and institutional ethical regulations. Protocols were approved by the Human Gamete, Embryo, and Stem Cell Research Committee (institutional review board) at the University of California, San Francisco. Primary brain tissue samples were sectioned perpendicularly to the ventricle to obtain slices 300 μm thick and ∼2.5 mm^2^ in surface area, using a Leica VT1200S vibrating blade microtome and artificial cerebrospinal fluid containing 125 mM NaCl, 2.5 mM KCl, 1 mM MgCl_2_, 1 mM CaCl_2_, and 1.25 mM NaH_2_PO_4_. Explants were transferred to slice culture insertions (Millicell) in 6-well culture plates and were cultured with media containing 66% Eagle’s basal medium, 25% Hanks balanced salt solution, 5% fetal bovine serum, 1% N-2 supplement, 1× penicillin-streptomycin, and glutamine in a 37°C incubator with 5% CO_2_. At 12 h after plating, slices were inoculated with 10^4^ B. mandrillaris trophozoites in 20 µl of complete media containing vehicle (DMSO) or 35 µM nitroxoline, which was added dropwise to the slice surface. Media below the cell culture insert was adjusted to matching vehicle or nitroxoline concentrations. At 20 h postinoculation, media below the cell culture insertion were replaced with fresh media containing no drug. At that time, amoebae on top of the insertion surrounding and within the tissue were left undisturbed. Bright-field and phase contrast images were captured during the live culture experiment at magnifications of ×4, ×10, and ×20 using an Evos FL Cell Imaging System. At 4 days postinoculation, slices were gently fixed in 3.7% paraformaldehyde (PFA) for 4 h at 4°C and then rinsed with phosphate-buffered saline (PBS), stained for 2 h at room temperature with DAPI (4′,6-diamidino-2-phenylindole) (0.3 µM)–PBS–1% Triton X-100, and then mounted with ProLong Gold antifade mountant (Thermo). Images of stained tissue were obtained with a Nikon Ti spinning disk confocal microscope at ×20 magnification. Confocal z-stacks were projected and adjusted in ImageJ (78). Bright-field images were stitched together using photomerge in Photoshop (Adobe). Bright-field time-lapse images were processed as movies in ImageJ.
