# Supplementary material for: Functional Assessment of 2,177 U.S. and International Drugs Identifies the Quinoline Nitroxoline as a Potent Amoebicidal Agent against the Pathogen Balamuthia mandrillaris
Source: mBio. 2018 Oct 30;9(5):e02051-18. doi: 10.1128/mBio.02051-18 (PMC6212833; doi:10.1128/mBio.02051-18)
Supplement: TABLE S2 [file mbo005184140st2.pdf]

Table S2

Compound name	Compound number	Structure	IC_{50} (μM)	R^2	Hill slope	Compound source
8-Hydroxy-5-nitroquinoline	**1**	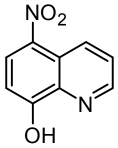	4.77	0.980	3.49	Selleck Chemical
8-Hydroxyquinoline	**2**	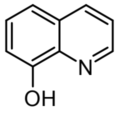	30.51	0.929	1.27	Sigma-Aldrich
5-Nitroquinoline	**3**	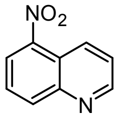	82.69	0.888	0.93	Sigma-Aldrich
5-Fluoroquinolin-8-ol	**4**	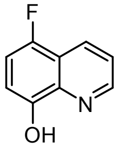	60.41	0.953	4.92	MolPort
5-Chloro-8-quinolinol	**5**	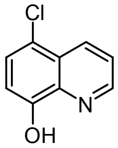	28.69	0.968	16.11	Sigma-Aldrich
5-Bromo-8-hydroxy quinoline	**6**	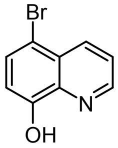	48.94	0.991	3.35	AK Scientific
5-Methylquinolin-8-ol	**7**	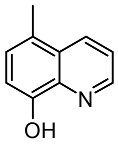	19.94	0.984	3.57	AK Scientific
5-Amino-8-hydroxyquinoline dihydrochloride	**8**	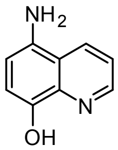	37.55	0.664	0.82	Sigma-Aldrich
5-Nitroso-8-hydroxyquinoline	**9**	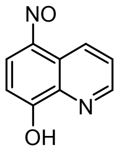	48.95	0.986	4.14	AK Scientific
5,7-Dichloro-8-quinolinol	**10**	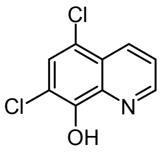	50.03	0.948	3.69	Sigma-Aldrich

Table S2 (continued)

Compound name	Compound number	Structure	IC_{50} (μM)	R^2	Hill slope	Compound source
5,7-Dinitro-8-quinolinol	**11**	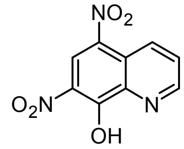	58.89	0.903	4.49	Sigma-Aldrich
8-Hydroxy-6-nitroquinoline	**12**	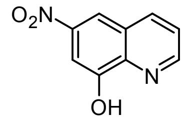	16.80	0.894	3.82	MolPort
8-Hydroxy-5-quinolinesulfonic acid	**13**	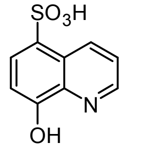	338.3	0.769	1.21	Sigma-Aldrich
1,10-Phenanthroline	**14**	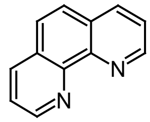	15.89	0.967	3.71	Sigma-Aldrich
6-Nitroquinolone	**15**	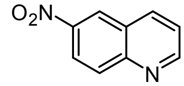	189.4	0.808	1.41	Sigma-Aldrich
8-Quinolinol N-oxide	**16**	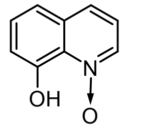	165.0	0.756	1.64	Sigma-Aldrich
8-Ethoxy-5-nitroquinoline	**17**	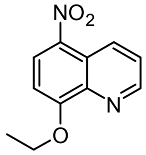	434.2	0.709	1.37	Sigma-Aldrich
2-Quinolinecarboxylic acid	**18**	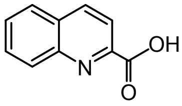	884.2	0.266	1.28	Sigma-Aldrich
3-Nitro-4-quinolinol	**19**	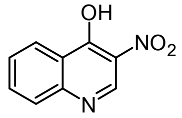	1324	0.315	1.07	Sigma-Aldrich
Quinoline	**20**	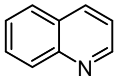	Not converged			Sigma-Aldrich

Table S2. Structures, IC_{50} values, and dose-response curve fitting parameters for nitroxoline and all nitroxoline analogs tested for activity against *B. mandrillaris*. IC_{50}, R^2, and Hill slope values were calculated based on sigmoidal curves fit to 8-point dose-response experiments performed in triplicate. Compounds 2, 4-12, and 14 were the most potent inhibitors of *B. mandrillaris*, most likely due to the presence of an 8-position hydroxyl group or bidentate ligand on the quinoline ring, which are predicted to be necessary for metal binding activity.
