# Supplementary material for: Functional Assessment of 2,177 U.S. and International Drugs Identifies the Quinoline Nitroxoline as a Potent Amoebicidal Agent against the Pathogen Balamuthia mandrillaris
Source: mBio. 2018 Oct 30;9(5):e02051-18. doi: 10.1128/mBio.02051-18 (PMC6212833; doi:10.1128/mBio.02051-18)
Supplement: TABLE S3 [file mbo005184140st3.pdf]

Table S3

	B. mandrillaris trophozoites	*B. mandrillaris* cysts	U87 cell line		H4 cell line		HEK-293T cell line		HFF-1 cell line		Hep-G2 cell line		Mean of all cell lines	
	IC_{50}[a]	IC_{50}[a]	CC_{50}[b]	SI[c]	CC_{50}[b]	SI[c]	CC_{50}[b]	SI[c]	CC_{50}[b]	SI[c]	CC_{50}[b]	SI[c]	CC_{50}[b]	SI[c]
**Nitroxoline**	2.8	15.5	24.9	8.77	5.6	1.96	4.9	1.71	51.8	18.23	9.3	3.29	19.3	6.79
**Pentamidine isethionate**	9.1	26.3	20.7	2.26	5.7	0.63	4.8	0.52	23.4	1.95	0.7	0.25	11.1	1.12
**Miltefosine**	63.2	76.5	34.7	0.55	8.4	0.15	68.8	1.21	67.1	1.10	66.1	0.94	49.0	0.79
**Azithromycin**	244.1	788.4	55.0	0.21	95.0	0.39	119.9	0.59	115.8	0.47	86.4	0.27	94.4	0.39

[a]IC_{50} = half-maximal inhibitory concentration
[b]CC_{50} = half-maximal cytotoxic concentration
[c]Selectivity Index (SI) = (cell line CC_{50})/(*B. mandrillaris* trophozoite IC_{50})

Table S3. Summary of IC_{50}, CC_{50}, and SI values calculated from dose-response experiments. IC_{50} and CC_{50} values were calculated from dose-response curves generated by measuring the viability of cultured cells 72 hours after drug treatment. Cytotoxicity of compounds was measured in U87, H4, HEK-293T, HFF-1, and Hep-G2 cell lines. All experiments were performed in triplicate. SI values for each drug show the ratio of the CC_{50} value for a given cell line to the IC_{50} value for *B. mandrillaris* trophozoites. An SI value greater than 1 indicates that the drug inhibits *B. mandrillaris* trophozoites at lower concentrations than the human cell line used for comparison (i.e. has positive selectivity). Nitroxoline is the most potent inhibitor (lowest IC_{50}) of both forms of *B. mandrillaris* and has the highest SI for all cell lines with a mean SI of 6.79.
