# Supplementary material for: Functional Assessment of 2,177 U.S. and International Drugs Identifies the Quinoline Nitroxoline as a Potent Amoebicidal Agent against the Pathogen Balamuthia mandrillaris
Source: mBio. 2018 Oct 30;9(5):e02051-18. doi: 10.1128/mBio.02051-18 (PMC6212833; doi:10.1128/mBio.02051-18)
Supplement: FIG S1 [file mbo005184140sf1.pdf]

Figure S1

A

B

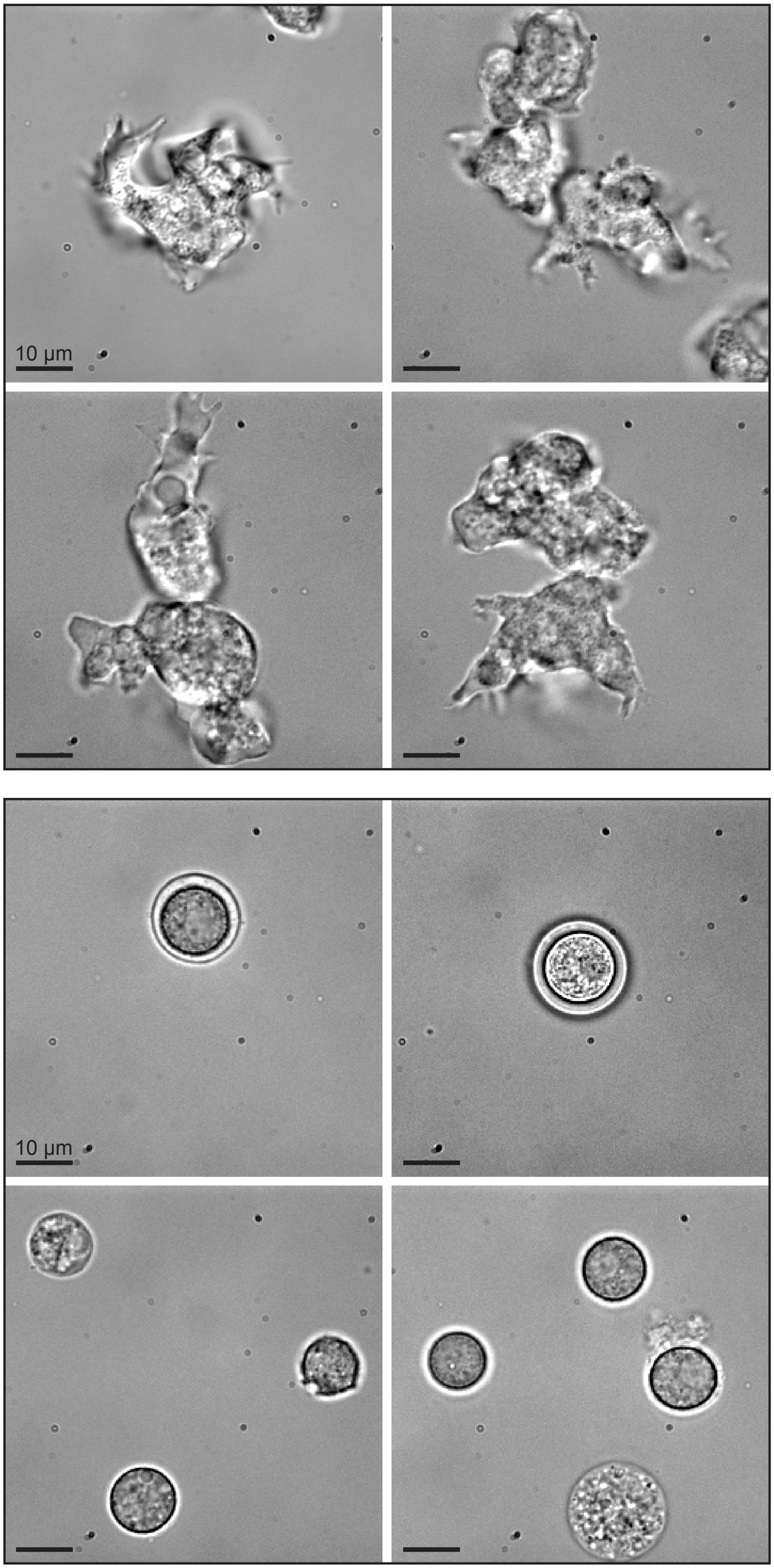

Figure S1. Example brightfield images of *B. mandrillaris* trophozoites and cysts. (A) Example images show *B. mandrillaris* trophozoites in log-phase growth. Trophozoites are pleomorphic and can be elongated or generally rounded, often with highly branched pseudopodia. (B) Example images show B. mandrillaris cysts induced by galactose exposure. Cysts are spherical, generally smaller in diameter than trophozoites, and can have visibly distinct layers. Some cysts show signs of vacuolization that may be indicative of cell death.
