# Supplementary material for: Functional Assessment of 2,177 U.S. and International Drugs Identifies the Quinoline Nitroxoline as a Potent Amoebicidal Agent against the Pathogen Balamuthia mandrillaris
Source: mBio. 2018 Oct 30;9(5):e02051-18. doi: 10.1128/mBio.02051-18 (PMC6212833; doi:10.1128/mBio.02051-18)
Supplement: FIG S2 [file mbo005184140sf2.pdf]

Figure S2

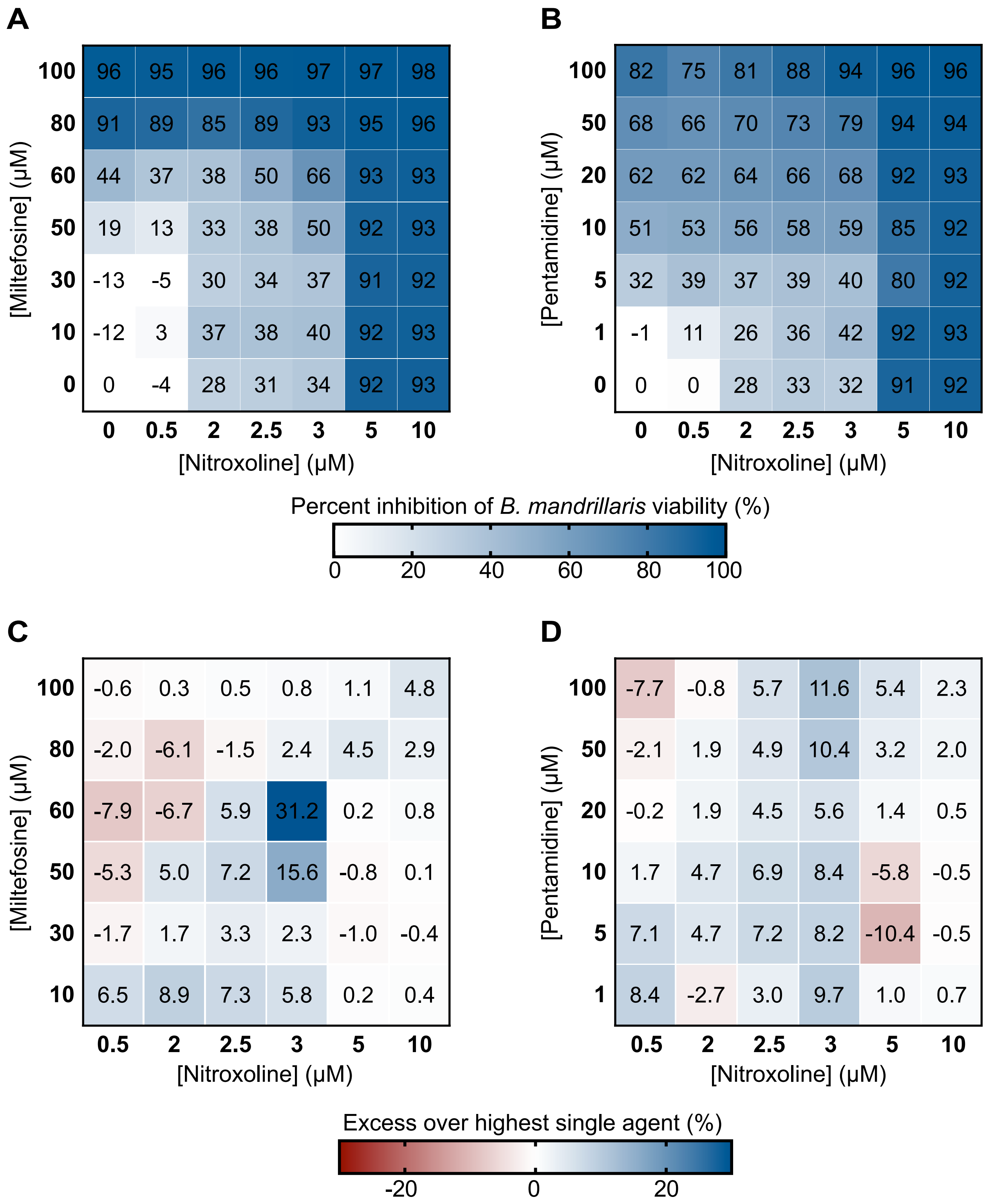

Figure S2. Efficacy of nitroxoline in combination with miltefosine and pentamidine isethionate for inhibition of *B. mandrillaris*. (A and B) Heat maps show mean treatment effect (percent inhibition of *B. mandrillaris* population viability relative to untreated control) across dose matrices for nitroxoline in combination with miltefosine (A) and pentamidine isethionate (B). The viability of *B. mandrillaris* trophozoite populations was measured following 72 hours of drug treatment in three independent replicates. (C and D) Heat maps of excess effect over highest single agent (EOHSA) showing the additional effect of dose combinations beyond the effect of the most active single agent in the combination (EOHSA = E_{combo} - E_{HSA}) across dose matrices for nitroxoline in combination with miltefosine (C) and pentamidine isethionate (D). For both drug pairs, mild antagonism (negative values colored in red) is observed for a small number of combinations, whereas most combinations produce a modest additive effect (positive values colored in blue). No strong antagonism or synergy is observed between nitroxoline and miltefosine or pentamidine isethionate.
