# Supplementary material for: Functional Assessment of 2,177 U.S. and International Drugs Identifies the Quinoline Nitroxoline as a Potent Amoebicidal Agent against the Pathogen Balamuthia mandrillaris
Source: mBio. 2018 Oct 30;9(5):e02051-18. doi: 10.1128/mBio.02051-18 (PMC6212833; doi:10.1128/mBio.02051-18)
Supplement: FIG S3 [file mbo005184140sf3.pdf]

Figure S3

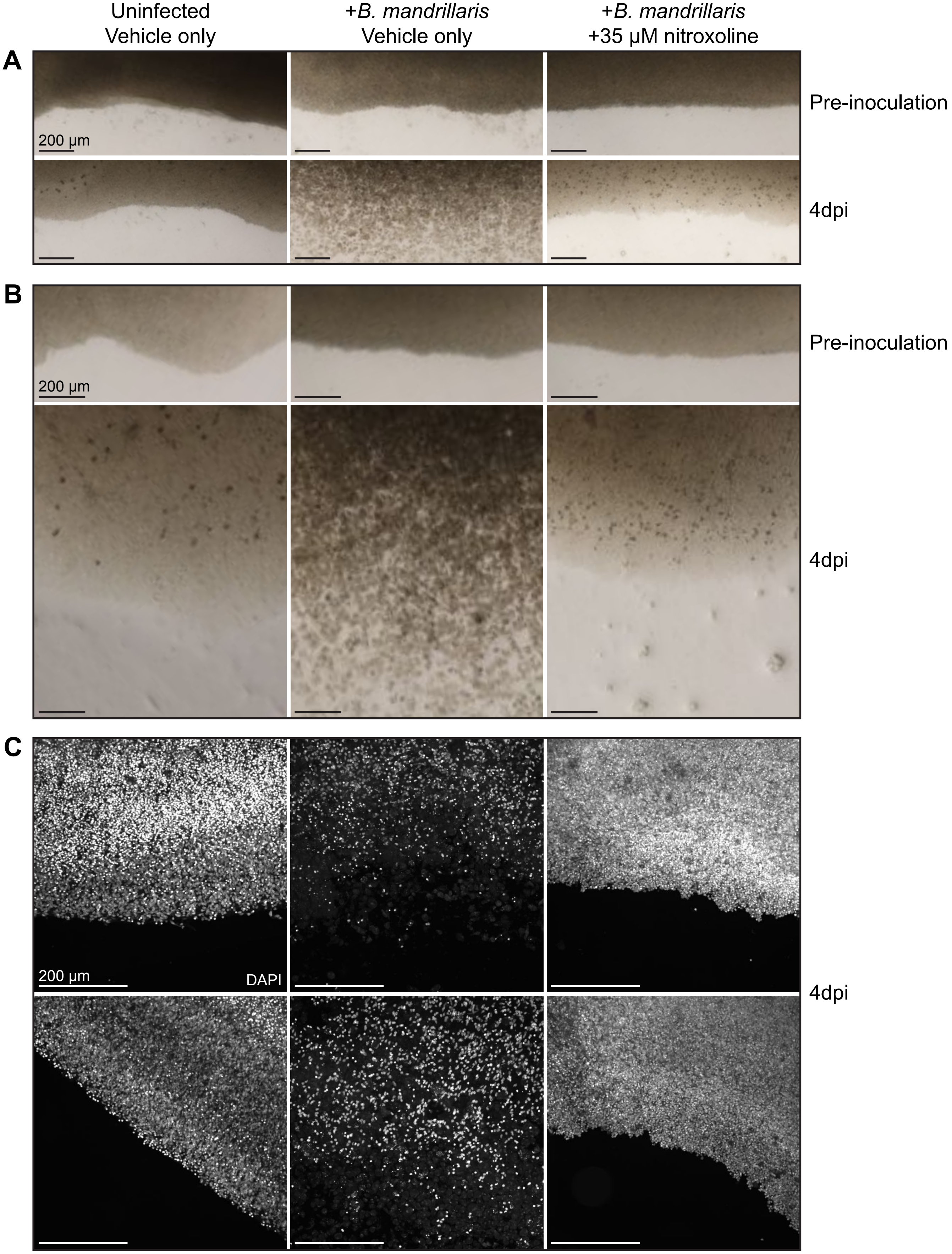

Figure S3. (A and B) Brightfield images representative of two biological replicates for human brain tissue explants before and after exposure to the indicated conditions. Nitroxoline or vehicle was added simultaneously with *B. mandrillaris* trophozoites and removed after 20 hours. Four days after exposure to *B. mandrillaris*, untreated tissues show widespread damage and loss of cell density while nitroxoline treated tissues remain intact and appear similar to uninfected tissues. Large numbers of *B. mandrillaris* trophozoites can be seen at the edges of untreated tissues, while only clusters of cysts are observed in nitroxoline-treated tissues. (C) Images representative of two biological replicates of human brain tissue explants fixed and stained with DAPI four days after exposure to the indicated conditions (two images per condition). The number of host cell nuclei is dramatically reduced in untreated, *B. mandrillaris*-infected tissues compared to uninfected tissues, whereas *B. mandrillaris*-infected tissues treated with nitroxoline show no apparent loss of nuclei.
